# HMGB1/RAGE induces IL-17 expression to exaggerate inflammation in peripheral blood cells of hepatitis B patients

**DOI:** 10.1186/s12967-015-0663-1

**Published:** 2015-09-21

**Authors:** JooYeon Jhun, SeungHoon Lee, HeeYeon Kim, Yang-Mi Her, Jae Kyeong Byun, Eun-Kyung Kim, Soon Kyu Lee, Mi-La Cho, Jong Young Choi

**Affiliations:** The Rheumatism Research Center, Catholic Research Institute of Medical Science, The Catholic University of Korea, Seoul, South Korea; Division of Rheumatology, Department of Internal Medicine, The Catholic University of Korea, Seoul, 137-040 South Korea; Division of Hepatology, Department of Internal Medicine, College of Medicine, Seoul St. Mary’s Hospital, The Catholic University of Korea, 505 Banpo-Dong, Seocho-Ku, Seoul, 137-040 South Korea; Conversant Research Consortium in Immunologic Disease, College of Medicine, The Catholic University of Korea, 505 Banpo-Dong, Seocho-Ku, Seoul, 137-040 South Korea

**Keywords:** HMGB1, RAGE, IL-17, Inflammation, Hepatitis B virus

## Abstract

**Background:**

Hepatitis B (HB) is an infectious disease with unfavorable consequence for patients and involved in chronic inflammation of liver. The present study aimed to investigate whether High-mobility group protein B (HMGB)1/receptor for advanced glycation end products (RAGE) aggravates inflammation enhancing the expression of interleukin (IL)-17.

**Methods:**

Mild and severe HB liver tissue and peripheral blood samples were obtained intra-operatively. Histological analysis of the livers was performed by immunohistochemistry. IL-1β and IL-6 of liver tissue were detected by confocal microscopy staining. Relative mRNA expression was measured by real-time PCR and protein levels were measured by enzyme-linked immunosorbent assay.

**Results:**

HMGB1, RAGE and IL-17 expression is increased in liver of HB patients with acute on chronic liver failure (ACLF) compared to healthy controls. HMGB1 treatment induced inflammatory cytokines including IL-17 in peripheral blood cells of HB patients. IL-17 also induced the expression of RAGE and IL-1β in peripheral blood cells of HB patients with ACLF. On the other hands, the inhibitory factor of p38 and nuclear factor-kappa B reduced the expression of RAGE and IL-1β in peripheral blood cells HB patients with ACLF.

**Conclusions:**

HMGB1, RAGE and IL-17 expression is increased in liver of severe HB patients. HMGB1 and RAGE interaction may contribute to the inflammation of liver enhancing the expression of IL-17, which can be possibly restored through the decline of the HMGB1/RAGE axis.

## Background

Patients infected hepatitis B virus (HBV) which is a major human pathogen can be chronic hepatitis B or asymptomatic carrier, and reveal chronic inflammation of the liver associated with HBV. But, abnormal liver functions or liver failure can be initiated during protracted term of chronic HBV infection [1]. Some of HBV related with acute on chronic liver failure (ACLF) can induce rapid outbreak of inflammation, sickness with vomiting, cirrhosis and high mortality [2, 3]. Additionally, several proinflammatory cytokines are involved in liver inflammation because HBV leads to inflammatory response in liver. For example, tumor necrosis factor (TNF)-α is related with chronic HBV infection [4]. Additionally, it has been reported that the levels of TNF-α, IL-1β and IL-6 are induced by HBV infection in a human hepatocyte model [5].

High-mobility group protein B (HMGB) 1 is a cytokine mediator of inflammation and secreted by immune cells such as macrophages and monocytes. It is well documented that HMGB1 was secreted in activated macrophages and monocytes inducing inflammatory response [6]. HMGB1 is involved in liver inflammation enhancing proinflammatory cytokine such as IL-17 expression, which induces neutrophil infiltration and inflammatory response of liver [7]. It has been suggested that the severity of chronic HB is correlated with upregulated HMGB1 levels in [8]. In addition, HMGB1 increases the synthesis of pro-IL-1β in macrophages by the activation of p38 mitogen-activated protein kinase (MAPK) and nuclear factor kappa B (NF-κB) [9].

The receptor for advanced glycation end products (RAGE), which is transmembrane protein of the immunoglobulin super family, functions as a receptor for molecular pattern recognition activating innate and adaptive immunity. This receptor interacts with HMGB1 to promote inflammation. It has been reported that HMGB1/RAGE axis enhanced inflammation [10]. Moreover, it has been demonstrated that RAGE mediated the activation of p38 MAPK and NF-κB and induced the secretion of proinflammatory cytokines [11, 12].

IL-17 is a proinflammatory cytokines expressed by T-helper cells and conducts a significant role in inflammation. For example, IL-17 results in inflammatory response through activation of p38 MAPK and NF-κB [13]. There are several evidences that the expression of IL-17 is involved in several inflammatory diseases [14–16]. As IL-17 initiates immune response through upregulating chemokine production in several tissues such as liver, the expression of IL-17 is involved in HB. For example, interleukin (IL)-17 producing CD4+ T cells are involved in liver inflammation and also increased in the bloods of HB patients pervading the liver tissues of HB patients [17, 18]. It is also well documented that the expression of IL-17 was promoted in serum of patients with chronic HB and correlated with the severity of fibrosis [19].

We hypothesized that IL-17, HMGB1 and RAGE are increased in liver of HB. The current study attempts to determine whether HMGB1/RAGE axis induces the expression of IL-17 through the activation of p38 MAPK and NF-κb in peripheral blood cells patients with HB. Thus, this research was carried out to elucidate that the upregulation of IL-17 expression induced by HMGB1 and RAGE exaggerates inflammation in peripheral blood cells of HBV-related acute on chronic liver failure (ACLF) patients.

## Methods

1. Patients and clinical information.
  We prospectively recruited eight patients with HBV-related ACLF at Seoul St. Mary’s Hospital (Seoul, Korea). Patients with comorbidities such as hepatitis C infection, human immunodeficiency virus infection, autoimmune liver diseases, hepatocellular carcinoma, alcoholic liver disease, and concurrent bacterial infection were excluded. Eight patients with HBV-related ACLF and eight healthy donors were age-matched and sex-matched, and then enrolled as controls for blood samples. The study was approved by the Institutional Review Board of Seoul St Mary’s Hospital (KC14SISI0008), and written informed consent was obtained from each subject. The patients were divided the severity of chronic hepatitis according to the Knodell Histology Activity Index Score [20]. Eight acute on chronic liver failure patients were enrolled in this investigation. Demographics of each acute on chronic liver failure patient group are listed in Table 1.

**Table 1:** Clinical characteristics of 8 acute on chronic liver failure patients

Number	Age	Sex	HBsAg	HBeAg	HBV DNA	AST	ALT	Albumin	Total bilirubin	INR
	(year)				log IU/ml	(U/L)	(U/L)	(g/dL)	(mg/dL)	
1	48	Female	positive	positive	5.6	147	250	2	4.4	7.27
2	59	Female	positive	negative	7.5	605	571	2.5	15.6	2.16
3	55	Female	positive	positive	6.1	112	342	3.43	5.8	3.04
4	44	Female	positive	positive	5.4	92	101	3.2	15.5	2.08
5	40	Male	positive	positive	4.7	93	135	2.9	27.1	3.15
6	47	Male	positive	positive	7.9	271	421	3.2	12.1	2.55
7	52	Male	positive	negative	8.1	581	1787	3.4	11.1	3.84
8	51	Male	positive	negative	7.1	350	490	3.4	10.9	3.31

*HBsAg* hepatitis B surface antigen, *HBeAg* hepatitis e antigen, *AST* aspartate aminotransferase, *ALT* alanine aminotransferase, *INR* international normalized ratio, *INR* international normalized ratio

2. Collection of peripheral blood mononuclear cells.
  Peripheral blood mononuclear cells were isolated from heparinized blood samples by Ficoll-Hypaque (GE Healthcare, Bioscience, Sweden, Uppsala) density-gradient centrifugation, The isolated cells were cultured. Cell cultures were performed in RPMI1640 medium (GibcoBRL, Carlsbad, CA, USA) containing penicillin (100 U/Ml), streptomycin (100 μ/ml) and 10 % fetal bovine serum (GibcoBRL, Carlsbad, CA, USA) that had been inactivated by heating to 55 °C for 30 min. The cell suspensions were dispensed into 48-well plates (Nunc, Roskilde, Denmark).

3. Cell treatment.
  Peripheral blood mononuclear cells isolated from heparinized blood samples were treated by NF-κB inhibitor (Sigma), IL-17 (R&D Systems), p38 and sRAGE (InvivoGen).

4. Measurement of cytokines.
  Cytokines concentration of IL-1β, IL-6, TNF-α and IL-17 in culture supernatants were analysis by ELISA. Antibodies directed against human IL-1β, IL-6, TNF-α and IL-17 and against biotinylated anti-human IL-1β, IL-6, TNF-α and IL-17 were used as the capture and detection antibodies, respectively.
  Alkaline phosphatase (Sigma) was used for the chromogenic reaction. The amounts of cytokines present in the test samples were determined from standard curves constructed with serial dilutions of recombinant human IL-1β, IL-6, TNF-α and IL-17 (R&D Systems, Minneapolis, MN, USA). The absorbance was determined with an ELISA microplate reader at 405 nm.

5. RNA extraction and real-time quantitative polymerase chain reaction.
  After incubation for 24 or 72 h with HMGB (R&D Systems, Minneapolis, MN, USA), mRNA was extracted using RNAzolB (Biotex Laboratories, Huston, TX, USA) according to the manufacturer’s instructions. Polymerase chain reaction (PCR) amplification and analysis were achieved using a LightCycler 2.0 instrument (Roche Diagnostic, Mannheim, Germany) with software version 4.0. All reactions were performed with the LightCyclerFastStart DNA Master SYBR green I (Takara, Shiga, Japan) according to the manufacturer’s instruction. The following primers were used: Human IL-17, 5′-CAA CCG ATC CAC CTC ACC TT-3′ (sense) and 5′-GGC ACT TTG CCT CCC AGA T-3′ (antisense); IL-6, 5′-TGC TCC TGG TGT TGC CTG CT-3′ (sense) and 5′-AGC CAC TGG TTC TGT GCC TGC-3′ (antisense); IL-1b, 5′-GGA CAA GCT GAG GAA GAT GC-3′ (sense) and 5′-TCG TTA TCC CAT GTG TCG AA-3′ (antisense); TNF-a, 5′-GCC TCT TCT CCT TCC TGA TCG T-3′ (sense) and 5′-CTC GGC AAA GTC GAG ATA GTC G-3′ (antisense); RAGE, 5′-GAC TCT TAG CTG GCA CTT GGA T-3′(sense) and 5′-GGA CTT CAC AGG TCA GGG TTA C-3′ (antisense). mRNA expression was normalized to β-actin expression.

6. Immunohistochemical staining.
  Liver tissue were fixed in 4 % paraformaldehyde and embedded in Paraffin. Then 7 μm sections were prepared. The sections were deparaffinized using xylene and dehydrated in a gradient of alcohols. Endogenous peroxidase activity was quenched with methanol 3 % H_2_O_2_. Immunohistochemistry was performed using the Vectastain ABC kit (Vector Laboratories, Burlingame, CA, USA). Tissue were incubated with the first primary antibody for IL-17 (Santacruz), HMGB (Cell signaling), RAGE (R and D systems), and isotype control overnight at 4 °C, and a biotinylated secondary linking antibody and a streptavidin peroxidase complex for 1 h. The final color product was developed using DAB chromogen (DAKO, Carpinteria, CA, USA).

7. Immunofluorescence analysis.
  A 100 μl aliquot of each sample was put into the appropriate well of a cytospin chamber (Thermo Scientific, Michigan, MI, USA) and was centrifuged art 800*g* for 3 min at 4 °C. To preserve the membrane-associated components and foreclose cytoplasmic staining, cells were fixed with methanol-acetone at −20 °C for 10 min. Immunofluorescence investigation were carried out using the PE-conjugated anti-IL-b and IL-6 (1:100; BD Bioscience) and 4′,6-diamidino-2-phenylindole (DAPI). The stained sections were analyzed using a Zeiss microscope (LSM 510 Meta; Carl Zeiss, Oberkochen, Germany) at 400× magnification.

8. Statistical analysis.
  Results were expressed as medians (interquartile range), Statistical comparison between two groups were made by a Mann–Whitney U test. A P < 0.05 was considered statistical significant. All statistical tests were performed using GraphPad Prism v.4.03 software (GraphPad Software, La Jolla, CA, USA).

## Results

**Fig. 1:**
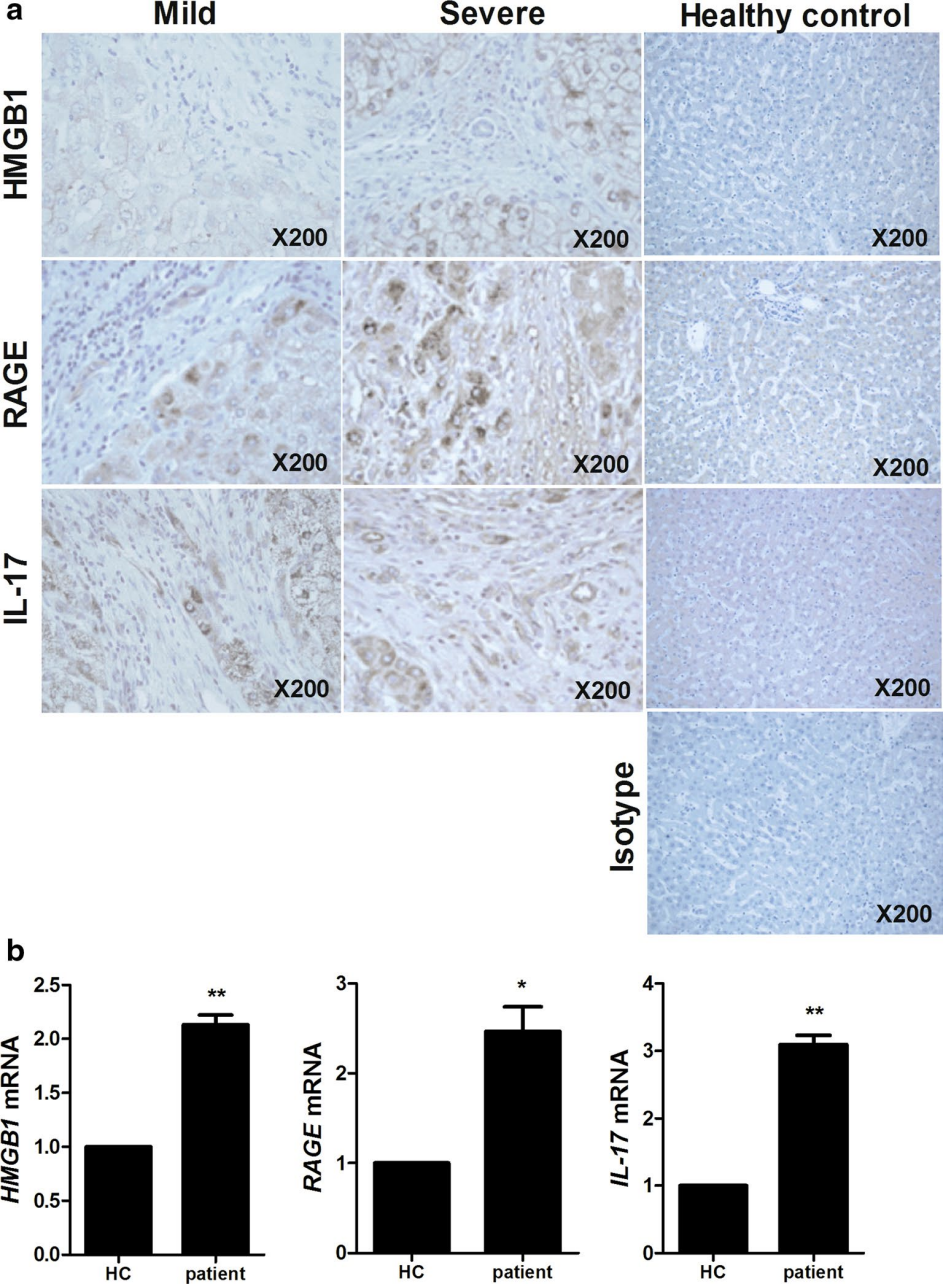
The expression of HMGB1, RAGE and IL-17 is positively related with severity of HB. **a** Histopathological analysis showed the expression of HMGB1, RAGE and IL-17 in liver of healthy controls, mild HB patients and severe HB patients. All tissues were counterstained with hematoxylin (original magnification, ×200). All images were obtained for each patients showing representative images. **b** The mRNA level of HMGB1, RAGE and IL-17 in peripheral blood cells of patients with HB was measured by realtime-PCR. Data are presented as the mean ± SD of three independent experiments (*p < 0.05, **p < 0.01)

**Fig. 2:**
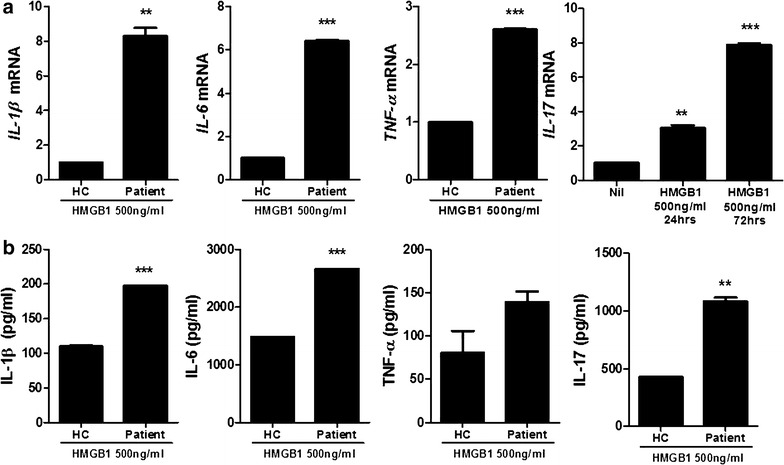
HMGB1 treatment promotes significantly the expression of proinflammatory cytokine in peripheral blood cells of HB patients compared to healthy controls. **a** The mRNA expression of IL-1β, -6, -17 and TNF-α in peripheral blood cells of patients with HB and healthy controls was measured by realtime-PCR (n = 3). **b** The protein level of IL-1β, -6, -17 and TNF-α in peripheral blood cells of patients with HB and healthy controls was measured by ELISA. Data are presented as the mean ± SD of three independent experiments (n = 3, **P < 0.03, ***P < 0.001)

**Fig. 3:**
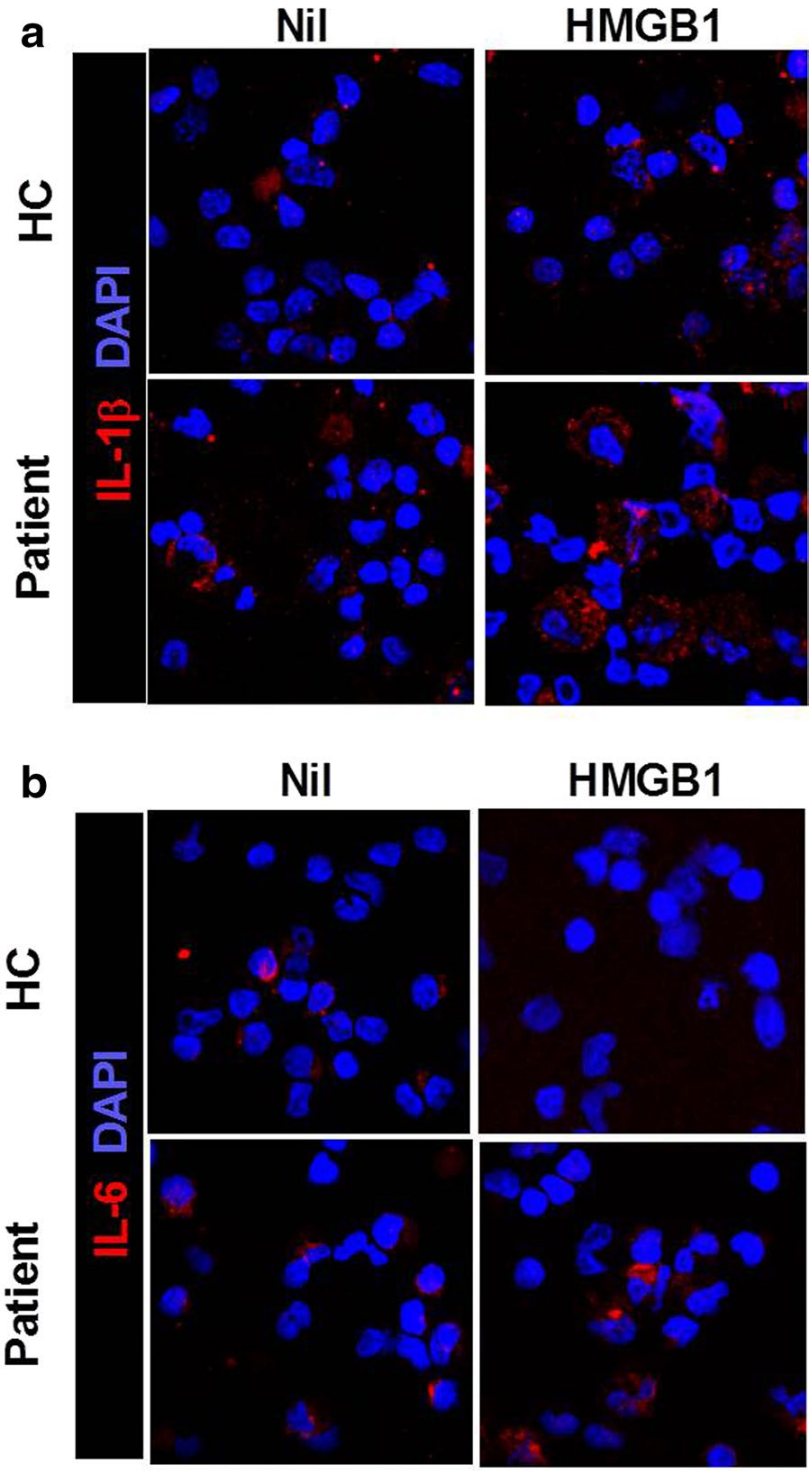
HMGB1 treatment more enhances the expression of proinflammatory cytokine in peripheral blood cells of HB patients than peripheral blood cells of healthy controls. Liver of HB patients or healthy controls as control were subjected to immunostaining for IL-1β and -6 (n = 3) (**a**, **b**)

**Fig. 4:**
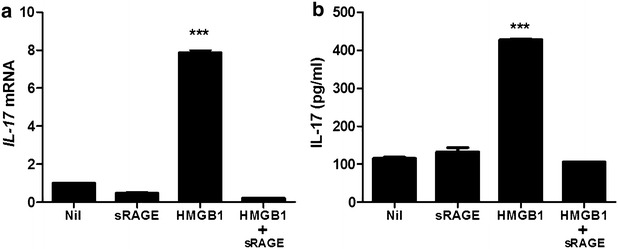
HMGB1 increases IL-17 expression through RAGE. The mRNA expression of IL-17 in peripheral blood cells of patients with HB was evaluated by realtime-PCR (**a**). The protein level of IL-17 in peripheral blood cells of patients with HB was evaluated by ELISA (**b**). Data are presented as the mean ± SD of three independent experiments (***p < 0.001)

**Fig. 5:**
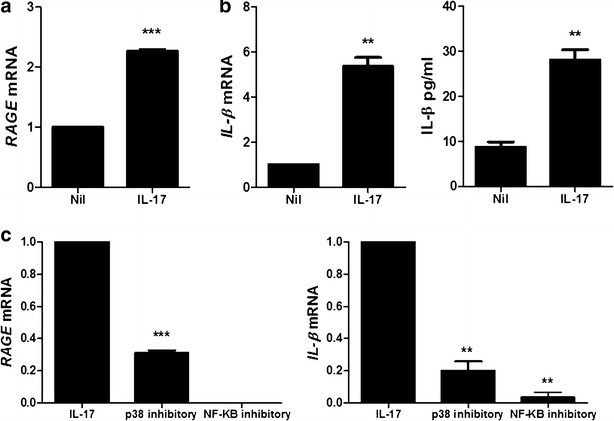
IL-17 promotes the expression of IL-1β and RAGE through p38 MAPK and NF-κB. **a** The mRNA expression of RAGE in peripheral blood cells of patients with HB was measured by realtime-PCR. **b** The mRNA expression and protein level of IL-1β in peripheral blood cells of patients with HB is measured by realtime-PCR and ELISA. **c** The mRNA expression of RAGE and IL-1β in peripheral blood cells of patients with HB was measured by realtime-PCR. Data are presented as the mean ± SD of three independent experiments (**P < 0.03, ***p < 0.001)

1. Histological expression of HMGB1, RAGE and IL-17 in the liver of patients of different groups.
  The expression of HMGB1, RAGE and IL-17 in liver tissues of HB patients was evaluated by histological analysis. These expressions were increased in liver tissues of severe HB patients compared to mild HB patients and healthy controls (Fig. 1a). Gene expression of HMGB1, RAGE and IL-17 was promoted significantly in peripheral blood mononuclear cells (PBMC) of HB patients (Fig. 1b).

2. The expression of proinflammatory cytokines induced by HMGB1 treatment in PBMC of healthy controls and HB patients.
  In order to determine whether HMGB1 can induce the inflammatory response in PBMCof healthy controls and HB patients, cells were cultured in the presence HMGB1. Real-time PCR was performed to investigate the effects of HMGB treatment (500 ng/ml) on the mRNA expression of proinflammatory (IL-1β, -6, -17 and TNF-α). The expression of proinflammatory cytokines in PBMC of HB patients were promoted significantly compared to healthy controls. Particularly, IL-17 production was increased time-dependently (Fig. 2a). Moreover, ELISA was used to measure the protein level of proinflammatory cytokines (IL-1β, -6, -17 and TNF-α) and these proinflammatory cytokines productions were increased significantly by HMGB1 treatment (500 ng/ml) compared to healthy controls (Fig. 2b).

3. Immunocytochemical expression of proinflammatory cytokines in PBMC of healthy controls and patients with HB.
  Confocal scanning revealed that IL-1β and -6 expressions were increased by HMGB1 treatment (500 ng/ml) in PBMC of HB patients (Fig. 3a, b). However, the expression of IL-1β and -6 in PBMC of healthy controls was not increased by HMGB1 treatment.

4. The expression of IL-17 induced by HMGB1 through RAGE.
  Real-time PCR and ELISA were performed to investigate the variation of IL-17 expression in PBMC of HB patients. The PBMC was cultured with HMGB1 to examine that HMGB1 can induce IL-17 expression. HMGB1 treatment (500 ng/ml, 72 h) increased significantly mRNA expression of IL-17 in PBMC of HB patients. However, IL-17 gene expression is not enhanced in PBMC cultured with HMGB1 and soluble form of the receptor for advanced glycation end products (sRAGE) (Fig. 4a). In ELISA, IL-17 production is upregulated by HMGB1 treatment (500 ng/ml, 72 h) (Fig. 4b). But, the expression of IL-17 is not increased by the treatment of HMGB1 and sRAGE.

5. Inflammatory response induced.
  To determine the induction of IL-1β and RAGE by IL-17, PBMC of HB patients was treated with IL-17 (10 ng/ml, 72 h). IL-17 treatment increased significantly the mRNA expression of RAGE and IL-1β in PBMC of HB patients. The expression of IL-1β in PBMC of HB patients was also promoted significantly by IL-17 (Fig. 5a, b). The inhibitory of p38 MAPK (10 μM, 72 h) and NF-κB (50 μM, 72 h) decreased markedly RAGE and IL-1β mRNA levels β in PBMC of HB patients (Fig. 5c).

## Discussion

It is generally believed that HMGB1, a significant chromatin protein interacting transcription factors, nucleosomes and histones, interacts with RAGE in inflammatory response and IL-17 cause inflammation through activation of p38 MAPK and NF-κB [13, 21–23]. However, there is a little evidence of the interaction of HMGB1/RAGE and IL-17 on inflammatory response and the mechanism of its action. Here, we studied the inflammatory activity of HMBG1/RAGE on inflamed peripheral blood cells through previously undiscovered mechanism.

The most meaningful observation in this study is reciprocal activity of HMGB1/RAGE and IL-17 in peripheral blood cells of HB patients. It has been demonstrated that the HMGB1/RAGE interaction may be pivotal in liver inflammation [24]. Several reports have indicated that IL-17 plays an important role in liver inflammation [25, 26]. But, the interaction of HMGB1/RAGE with IL-17 has been not investigated in liver inflammation with HB. In this investigation, we revealed the inflammatory function of HMGB1/RAGE inducing IL-17 production in peripheral blood cells of patients with HB.

Previous evidences have documented that HMGB1, RAGE and IL-17 are involved in liver inflammation [27–29]. We observed that the expression of HMGB1, RAGE and IL-17 in liver of severe HB patients is higher than these expressions in liver of mild HB patients. These results suggest that the expression of HMGB1, RAGE and IL-17 is positively related with severity of HB.

Several proinflammatory cytokines are involved in HB pathogenesis. It is well reported that IL-1β, -6 and TNF-α were increased in serum of HB patients [30]. In this study, HMGB1 treatment enhanced the gene expressions and protein levels of these cytokines in peripheral blood cells of HB patients compared to healthy controls. Thus, HMGB1 can aggravate inflammatory response in PB patients.

Even though HMGB1 and IL-17, inducer of inflammation, are involved in liver inflammation, there is a little evidence of the expression of IL-17 induced by HMGB1. Indeed, IL-17 expressing cells such as helper T cells and neutrophils are located in liver and involved in inflammatory liver disease [31, 32]. HMGB1 and RAGE are also expressed in human liver cells including Hepg2 cells and related with liver disorders such as hepatic injury and liver ischemia [24, 27, 33, 34]. In this investigation, IL-17 expression was promoted by HMGB1 treatment in peripheral blood cells of patients with HB. We also observed that HMGB1 leads to increase the expression of IL-17 through RAGE.

NF-κB, a key regulator in the immune response, and p38 MAPK are involved in inflammation. The activation of NF-κB and p38 MAPK enhanced the expression of inflammatory cytokine and is related with several inflammatory diseases including HB [35–37]. The noticeable finding is that IL-17 induces the mRNA level of RAGE and IL-1β expression and the inhibitor of p38 MAPK and NF-κB suppressed the mRNA expression of RAGE and IL-1β in peripheral blood cells of patients with HB. On basis of these results, we presumed that IL-17 may cause the expression of RAGE and IL-1β by the activation of p38 MAPK and NF-κB.

Although HMGB1 is potential to induce IL-17 expression and exaggerates HB, in vivo animal investigations are needed to confirm the inflammatory effect of HMGB1 treatment. In vivo animal studies conducted in HB model are required to further proof that HMGB1 results in the exaggeration of HB enhancing IL-17 expression. Moreover, in vitro assays covering upregulation of proinflammatory cytokines through HMGB1 treatment were performed using relatively small number of samples and, thus, showed the pilot data. However, this investigation is the first research to report and propose the possible pathogenic potential of attenuation of HMGB1 activity in HB patients with ACLF. Future studies with the large number of cases and in vivo animal experiments are thought to be required to verify our hypothesis more precisely.

The function of HMGB1 has been little studied in inflammatory response mediated by IL-17. The suppression of HMGB1 production established in this investigation indicates that HMGB1 promotes IL-17 expression and inflammation in HB. Our results demonstrate that the inhibition of HMGB1/RAGE interaction can reduce inflammation in HB. This prior investigation about HMGB1 inducing IL-17 suggests that HMGB1 can be strong therapeutic target in HB.

## Conclusions

The function of HMGB1 has been little studied in inflammatory response mediated by IL-17. The suppression of HMGB1 production established in this investigation indicates that HMGB1 can promote IL-17 expression and inflammation in HB. Our results demonstrate that the inhibition of HMGB1/RAGE interaction can reduce inflammation in HB. This prior investigation about HMGB1 inducing IL-17 suggests that HMGB1 can be strong therapeutic target in HB.
